# *H* *omo sapiens*, industrialisation and the environmental mismatch hypothesis

**DOI:** 10.1111/brv.70094

**Published:** 2025-11-07

**Authors:** Daniel P. Longman, Colin N. Shaw

**Affiliations:** ^1^ School of Sport, Exercise and Health Sciences Loughborough University Epinal Way Loughborough LE113TU UK; ^2^ Department of Evolutionary Anthropology University of Zurich Winterthurerstrasse 190 Zurich 8057 Switzerland

**Keywords:** *Homo sapiens*, hominins, mismatch, habitat, industrialisation, environment, adaptation, fitness, evolution, biological anthropology

## Abstract

For the vast majority of the evolutionary history of *Homo sapiens*, a range of natural environments defined the parameters within which selection shaped human biology. Although human‐induced alterations to the terrestrial biosphere have been evident for over 10,000 years, the pace and scale of change has accelerated dramatically since the onset of the Industrial Revolution in the late 18th century. Industrialisation has profoundly transformed our various natural habitats, driving rapid urban expansion, increasing reliance on fossil fuel energy and causing environmental contamination, ecosystem degradation and biodiversity loss. Today, most of the world's population resides in highly industrialised urban areas. These new primary human habitats differ fundamentally from our ancestral natural habitats, creating novel environmental challenges while, simultaneously, lacking key natural features linked to health and function. Although the adaptive capacity of humans has enabled survival in diverse and fluctuating environmental conditions, this capacity is limited. It is possible that the rapid industrialisation of our habitat is outpacing our adaptive capacity and is imposing selective pressures that threaten our evolutionary fitness. A growing body of observational and experimental evidence suggests that industrialisation negatively impacts key biological functions essential for survival and reproduction and, therefore, evolutionary fitness. Specifically, environmental contamination arising directly from industrial activities (e.g. air, noise and light pollution, microplastic accumulation) is linked to impaired reproductive, immune, cognitive and physical function. Chronic activation of the stress response systems, which further impairs these biological functions, also appears more pronounced in industrialised areas. Here, we consider whether the rapid and extensive environmental shifts of the Anthropocene have compromised the fitness of *Homo sapiens*. We begin by contrasting contemporary and ancestral human habitats before assessing the effects of these changes on core biological functions that underpin evolutionary fitness. We then ask whether industrialisation has created a mismatch between our primarily nature‐adapted biology and the novel challenges imposed by contemporary industrialised environments – a possibility that we frame through the lens of the Environmental Mismatch Hypothesis. Finally, we explore experimental approaches to test this hypothesis and discuss the broader implications of such a mismatch.

## I. INTRODUCTION

Over the last ~6–7 million years, the hominin lineage has successfully adapted to diverse climatic and ecogeographical challenges. Throughout the last 100,000 years, the remarkable capacity of *Homo sapiens* to adapt, both biologically and culturally, enabled the colonisation of nearly all terrestrial environments on Earth (Wells & Stock, 2007). However, contemporary humans now face unprecedented environmental challenges resulting directly from the rapid and extensive habitat changes caused by industrialisation. While our species has demonstrated significant adaptive capacity in the past, emerging evidence suggests that these novel and accelerated challenges may surpass our ability to adapt, thereby threatening our long‐term viability.

Although human‐induced alteration of the terrestrial biosphere has been evident for over 10,000 years, the pace and scale of change has accelerated dramatically since the onset of the Industrial Revolution in the late 18th century (Ellis, 2011) and the Great Acceleration that began in the 1950s (see Section II.4) (Steffen *et al*., 2011). While industrialisation has provided significant benefits (Glaeser, 2011), it has also driven a profound transformation of various natural habitats (Ellis, 2011). The global spread of industrialisation has necessitated the expansion of urban centres, croplands, pastures and plantations and the transformation of forests, waterways and air *via* the increased consumption of energy, water and fertilisers (Foley *et al*., 2005). The sheer scale of these changes is exemplified by the dramatic rise in global energy consumption, largely driven by fossil fuels, which, since 1950, has exceeded the total energy expenditure of the previous ~12,000 years (Syvitski *et al*., 2020). Deforestation and industrial agriculture alone have reduced the carbon storage potential of terrestrial ecosystems by over 50% (Erb *et al*., 2018). These land‐use changes are the primary drivers of the ongoing sixth mass extinction, marked by substantial global biodiversity loss (Dirzo *et al*., 2014; Ceballos *et al*., 2015). These modifications now threaten ecosystem functions essential for human biological function and survival, including food production, freshwater regulation, climate stability and disease mitigation (Rockström *et al*., 2009). The 21st century is characterised by an increasingly human‐dominated planet (Vitousek *et al*., 1997) – so much so that this has led to the recognition of a new geological epoch: the Anthropocene (Steffen *et al*., 2015).

Given the unprecedented speed, magnitude and recency of environmental change, a critical question emerges: is the ongoing industrial transformation of the various natural habitats of *Homo sapiens* impairing our evolutionary fitness (the ability to survive and reproduce)? We approach this question in a stepwise fashion. First, we compare 21st century human habitats with those of our ancestors over the past 5 million years (Section II). Next, we examine how these habitat changes affect core aspects of human biology that underpin evolutionary fitness (Section III). We then introduce the Environmental Mismatch Hypothesis, which posits that there may be a misalignment between the contemporary habitats of *Homo sapiens* and the adaptations that have primarily shaped the human phenotype (Section IV). Finally, we explore experimental approaches to test this hypothesis (Section V) and discuss its real‐world implications (Section VI).

## II. ENVIRONMENTAL CHANGE

This section examines how the environmental contexts – and associated selective pressures – of *Homo sapiens* and their ancestors has changed over the past ~5 million years. We begin by exploring humanity's current habitat in the 21st century, before tracing the environmental changes of previous epochs that culminated in the present.

### (1) 21st century: an industrialised continuum

In the 21st century, virtually every location on the planet has been impacted, to some degree, by human‐driven industrial processes. As a result, contemporary human habitats exist on an industrialised continuum, ranging from non‐industrialised ecosystems to fully industrialised environments. The former, widespread until ~12 thousand years ago (Kya) before the advent of agriculture, is now rare, with only a few remote locations, such as parts of the Amazon rainforest, remaining near this end of the continuum (Goudie, 2018). However, even these areas are likely influenced by industrial processes. For example, microplastic debris is now essentially ubiquitous throughout the planet (Rillig, 2012). At the opposite end of the continuum are modern megacities, such as Tokyo or Delhi, which are home to more than 10 million people and are characterised by the near‐complete absence of natural features (i.e. those shaped by ecological processes, not involving human manufacture) and an abundance of human‐manufactured structures made from concrete, steel, plastic and other highly processed materials (Bettencourt, 2013; Elhacham *et al*., 2020). In 2024, there were 32 megacities worldwide, six of which housed over 25 million people (Melchiorri *et al*., 2025).

All *Homo sapiens* living in the 21st century inhabit environments situated along this continuum. Industrial attributes are found in primarily natural environments, just as natural attributes appear in highly industrialised areas. Urban parks, for example, combine natural features like trees, soil and wildlife with the industrialised city context (Groenewegen *et al*., 2006). Similarly, most homes and indoor workplaces are constructed primarily of industrial materials but also include natural attributes like plants and gardens. Consequently, the 21st century habitats of *Homo sapiens* are not binary (i.e. industrialised or non‐industrialised) but are a blend of industrial and natural attributes. However, most humans now reside in areas significantly closer to the highly industrialised urban end of the spectrum, far from pristine natural environments (Steffen, Crutzen & McNeill, 2007).

**Fig. 1:**
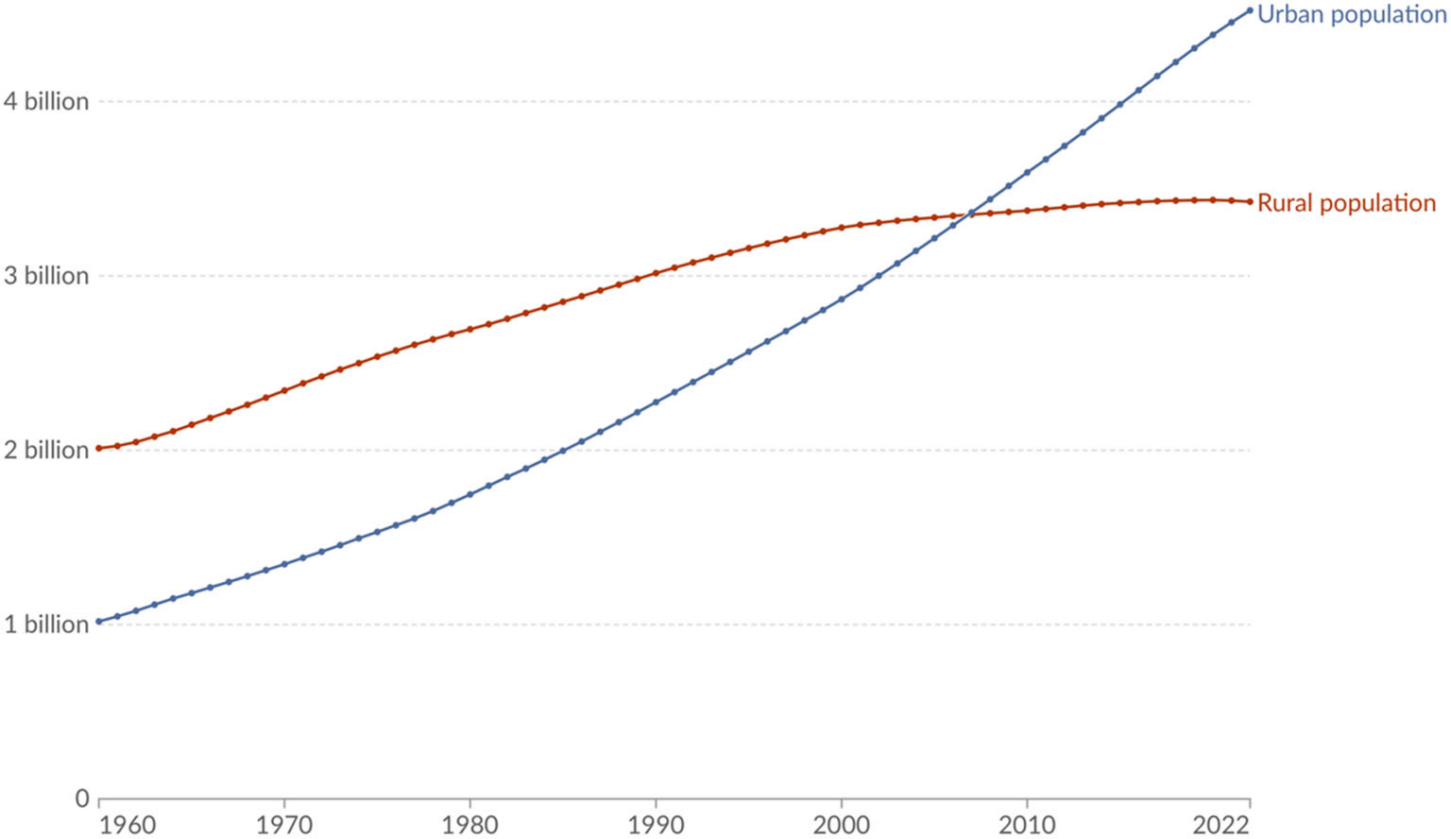
Temporal change in the global distribution of urban *versus* rural populations, 1960–2022. Taken from Ourworldindata.org (Ritchie *et al*., 2024).

According to the United Nations (UN), by 2018 over half (55%) of the world's population resided in urban areas, with this figure projected to rise to 68% by 2050 (Fig. 1) (United Nations, 2018). This global urbanisation is associated with a significant reduction in time spent outdoors, with people in countries like Canada (Matz *et al*., 2014), the USA (Klepeis *et al*., 2001), New Zealand (Khajehzadeh & Vale, 2017) and the UK (Baczynska, Khazova & O'Hagan, 2019) now spending ~93% of their day indoors. Consequently, *Homo sapiens* – especially in urban settings – has become an indoor‐urban species.

In the following sub‐sections, we look back to consider the Pliocene and Pleistocene habitats of our earliest hominin ancestors before moving forward in time to the Holocene and Anthropocene to consider the magnitude and rate of habitat change that has occurred throughout human evolutionary history.

### (2) Plio‐Pleistocene (~5.3 Mya – 12 Kya)

The Pliocene (5.3–2.6 million years ago, Mya) and Pleistocene (2.6 Mya – 12,000 Kya) epochs span ~5‐million years of our hominin ancestors' evolutionary journey. This is the period within which the majority of hominin fossil remains are found, although the earliest hominin fossil (*Sahelanthropus tchadensis*) is dated to 6–7 Mya (Brunet *et al*., 2002). During this time, long‐term feedback shaped both phenotypic and cultural adaptation to ecological challenges. These adaptations led to the development of the earliest stone tools at 3.3 Mya (Quinn *et al*., 2021), ubiquitous use of simple stone tools (the Oldowan) by 2 Mya (Toth & Schick, 2018), more complex stone tool technologies (de la Torre, 2016), and widening geographic spread (Carotenuto *et al*., 2016) between 1.75 and 1 Mya, complex hunting and materiality (Hutson *et al*., 2025) by 200 Kya, art and increasingly complex multi‐community social networks (Tylén *et al*., 2020) by 100 Kya and plant and animal domestication (Pinhasi & Stock, 2011) by 12–10 Kya.

These evolutionary milestones almost certainly tracked climatic conditions. For example, early hominins such as *Australopithecus* and *Paranthropus* primarily inhabited wooded, well‐watered regions, while the gradual shift from closed to open habitats between 4 and 2 Mya coincided with adaptations that led to the emergence of the genus *Homo*, the first hominins to exist in open, arid grasslands (Reed, 1997). Later, the controlled use of fire at ~1.5 Mya extended waking hours, influenced circadian rhythms, extended dietary possibilities, reduced digestion costs and allowed for dispersal into cooler regions (Attwell, Kovarovic & Kendal, 2015) (although note that these early dates for the habitual use of fire remain debated; Roebroeks & Villa, 2011).

Throughout this long period of time, hominins inhabited environments composed of materials (e.g. soil, plants and rocks) produced through natural geological and organic processes. Their impact on these environments was minimal. For instance, the production of tools from stone, wood and bone, among the most complex technologies of the time, required little processing. Overall, the environmental impact of hominins during this ~5‐million‐year period was minimal compared to later *Homo sapiens* populations.

Paleoenvironmental reconstructions of hominin sites have provided valuable insight into the environmental contexts that influenced selection and adaptation. Early hominins such as *Ardipithecus* (~4.4 Mya) were associated with woodland habitats, while *Australopithecus* (~4.5–2.0 Mya) inhabited diverse landscapes including woodlands, forests, riparian areas and grasslands (Behrensmeyer & Reed, 2013; Curran & Haile‐Selassie, 2016). Similarly, early *Homo* and *Paranthropus* occupied heterogeneous environments, ranging from woodlands to dense bushlands (Plummer *et al*., 2015).

Palaeosol analysis from Kenya's Turkana Basin reveal a consistent trend of aridification over the past 4.3 million years. The last period of heightened aridity, which occurred between 1.8 and 1.6 Mya, coincided with the emergence of early *Homo erectus* in Africa (Wood & Strait, 2004; Wynn, 2004). Paleoreconstructions of Dmanisi in Georgia (~1.9–1.8 Mya) suggest a climate resembling the present‐day Mediterranean, while the Sima del Elefante hominin site (1.2–0.8 Mya) in Spain indicates a forested environment (Blain *et al*., 2014; Hardy *et al*., 2017). These findings, along with analyses of similar sites in the UK and East Asia, suggest that dispersals outside Africa depended on favourable environments such as forested river margins and temperate forest mosaics (Parfitt *et al*., 2010; Zeller *et al*., 2023). European Neanderthal sites from the Mid‐ to Late‐Pleistocene (~250–40 Kya) suggest that they were similarly well adapted to forest and grassland habitats (Henry, Brooks & Piperno, 2011; Stewart *et al*., 2019).

The earliest evidence of *Homo sapiens* dates to approximately 300 Kya (Hublin *et al*., 2017). Around 100 Kya, *Homo sapiens* ventured out of Africa and dispersed widely across the Middle East, Central, South and Southeast Asia, Melanesia, Australia and eventually the Americas (Groucutt *et al*., 2015; Hershkovitz *et al*., 2018). Throughout this period, *Homo sapiens* inhabited a wide range of ecological extremes including deserts (Groucutt & Petraglia, 2012; Blinkhorn *et al*., 2017), high‐altitude regions (Beall, 2013), the Palaeoarctic (Pitulko *et al*., 2016) and tropical rainforests (Roberts *et al*., 2016).

### (3) Holocene (12 Kya – 300 years ago)

The Holocene Epoch covers approximately 12,000 years of the human evolutionary journey. The agriculture revolution began at the start of the Holocene and is perhaps the single most significant social, cultural and biological transition since the origin of our species. This transition enabled humans to exert control over the reproduction and evolution of plants and animals and marks a shift from lives based exclusively around the hunting, gathering and collecting of wild animals and plants to agriculture and the domestication of wild plants and animals. Humans shifted from being subject to changes in the natural environment to becoming the agents of environmental change in which the natural world was modified to suit human needs (Pinhasi & Stock, 2011; Ellis *et al*., 2021). From an evolutionary perspective, the process of plant and animal domestication was exceptionally fast, first originating in a dozen independent centres before spreading rapidly. The Holocene is associated with marked population growth globally, suggesting that agriculture may have spread because it was a behavioural strategy that promoted reproduction (Pinhasi & Stock, 2011).

While humans still inhabited environments predominantly composed of natural materials, agricultural practices initiated a significant change. This transition, which began at different times and in different geographic locations, generated agricultural surpluses, leading to the rise of larger communities, intensive land use and increasing sedentism (Pinhasi & Stock, 2011; Stephens *et al*., 2019). The earliest evidence for agriculture dates to the Levantine ‘Natufian’ period (14,500–11,600 years before present, BP), and is associated with the exploitation of wild grains, grindstones, stone architecture and organised site structures (Bar‐Yosef, 1998). The first evidence for larger human settlements with permanent architecture and intensive use and storage of grains in the Levant appears around 11,000 BP (Kuijt & Finlayson, 2009). While the transition to agriculture was burgeoning by 9000 BP (Twiss, 2007), hunting and gathering was also still practiced (Rosen & Rivera‐Collazo, 2012). Globally, agriculture spread at different rates depending on geographic and climatic conditions, reaching Southern Europe between 8000 and 7500 BP (Zeder, 2008) and Northern Europe by ~4000 BP (Jones *et al*., 2017; Bellwood, 2023). While population growth in pre‐agricultural populations likely occurred at a very slow rate due to local boom–bust dynamics (Shennan, 2002), the transition to agriculture was associated with more systematic population growth (Armelagos, Goodman & Jacobs, 1991; Bocquet‐Appel, 2011). This growth is exemplified by the emergence of villages and urban settlements in the Levant, China and West Africa between 12,000 and 5000 BP (Fuller *et al*., 2015).

The rise of food production and surplus storage that began with the origins of agriculture fuelled innovative cultural changes including property ownership, social hierarchy, task specialisation and runaway technological evolution (Ellis, 2011). As agricultural practices matured, they catalysed a complex restructuring of societal hierarchies and roles. The agricultural surplus generated through innovative techniques developed during this period enabled specialisation beyond subsistence farming and gave rise to a diverse range of professions (Weisdorf, 2005). The technological and cultural developments that grew out of this diversification paved the way for the Bronze (~3300–1200 BP) and Iron (~1200–550 BP) ages, characterised principally by the advent of bronze and steel tools and the development of increasingly complex urban societies (Molloy, 2023). By 1700, many societies had fully integrated these agricultural advancements into their social fabric and had paved the way for feudal systems that were deeply rooted in agrarian practices, land ownership and serfdom (Wallerstein & Wallerstein, 2021). The intricate tapestry of human development created during the Holocene laid the groundwork for the next phase, an even more impactful global cultural revolution.

### (4) Anthropocene (~300 years ago to present)

Over the past 200–300 years, human actions have become the main driver of global environmental change, with the rate of change accelerating dramatically after the mid‐20th century (Rockstrom *et al*., 2009; Waters *et al*., 2016). During the Holocene, the Earth's regulatory capacity maintained conditions that enabled extensive human development. By contrast, the relatively recent, large‐scale expansion of industrial processes throughout the globe – epitomised by humanity's reliance on fossil fuels, industrial agriculture and extensive resource extraction – has created conditions that are increasingly hostile to human life (Rockstrom *et al*., 2009).

**Fig. 2:**
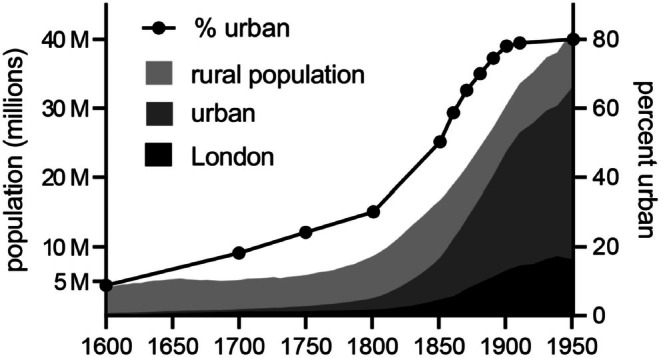
Urban population growth in England, 1600–1950. Adapted from Davenport (2021).

Commencing in late 1700s Britain, the Industrial Revolution marked a significant milestone in the evolutionary journey of *Homo sapiens*. This period witnessed a profound shift from agrarian to industrial societies, driven by the mechanisation of agriculture and the subsequent migration of former farm labourers to cities where factory jobs were plentiful (Faulkner, 1931). During the 1800s, these rural migrants fuelled the growth of the largest number of new cities in human history as well as rapid population growth in existing cities (Fig. 2). Manchester's population, for example, increased sixfold from 1771 to 1831 (Kidd, 2006), while London's grew from around 1 million people in 1800 to over 6.5 million people by 1900 (Dennis, 2022). The rapid growth of cities often led to overcrowding, inadequate sanitation and public health crises, exemplified by the 1832 and 1854 cholera outbreaks in London (Thomas, 2015). While the process of urbanisation was mainly limited to the developed world during the first half of the 1900s, developing countries witnessed spectacular growth in their urban populations during the latter half of the century (Mohan & Dasgupta, 2005).

Factory work epitomised the urban shift of the 1800s (Mantoux, 1929). As demand for industrial raw materials surged, deforestation and land conversion ensued, transforming untouched landscapes into cultivated fields and urban spaces (Foley *et al*., 2005). Exceptional increases in coal consumption led to unprecedented air pollution in urban centres (Akatsu, 2015). Discharge of industrial chemicals and untreated human waste into urban rivers resulted in polluted water systems (Scott & Baltzly, 1930). Despite rapid economic growth, living standards generally deteriorated and morbidity and mortality increased until the early 20th century (Voth & Leunig, 1996; Davenport, 2021).

**Fig. 3:**
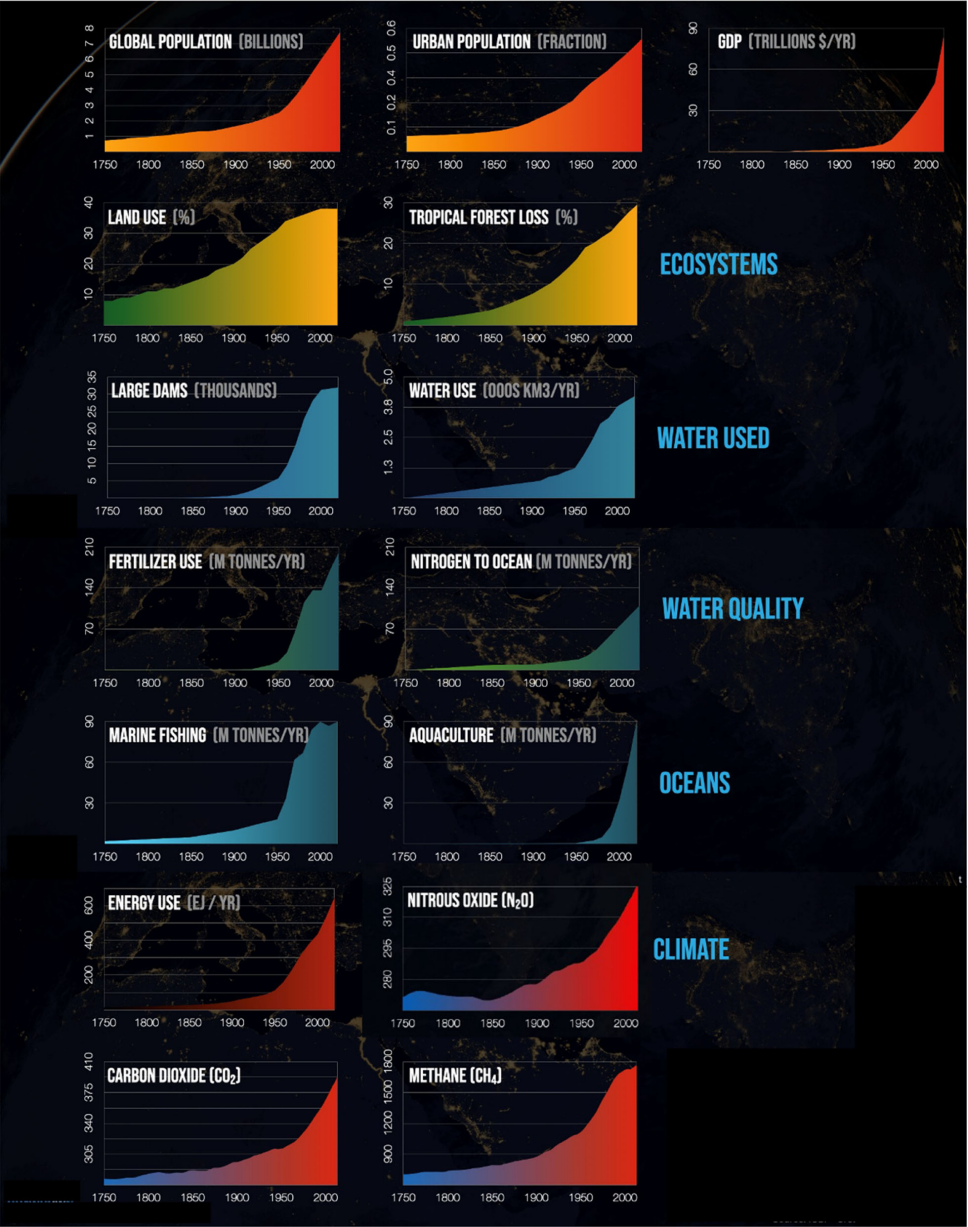
The Great Acceleration. Changes in human activity from 1950 onwards is reflected by a fundamental shift in the state and functioning of the Earth's natural systems. GDP, Gross Domestic Product. © 2021 Project Drawdown.

**Fig. 4:**
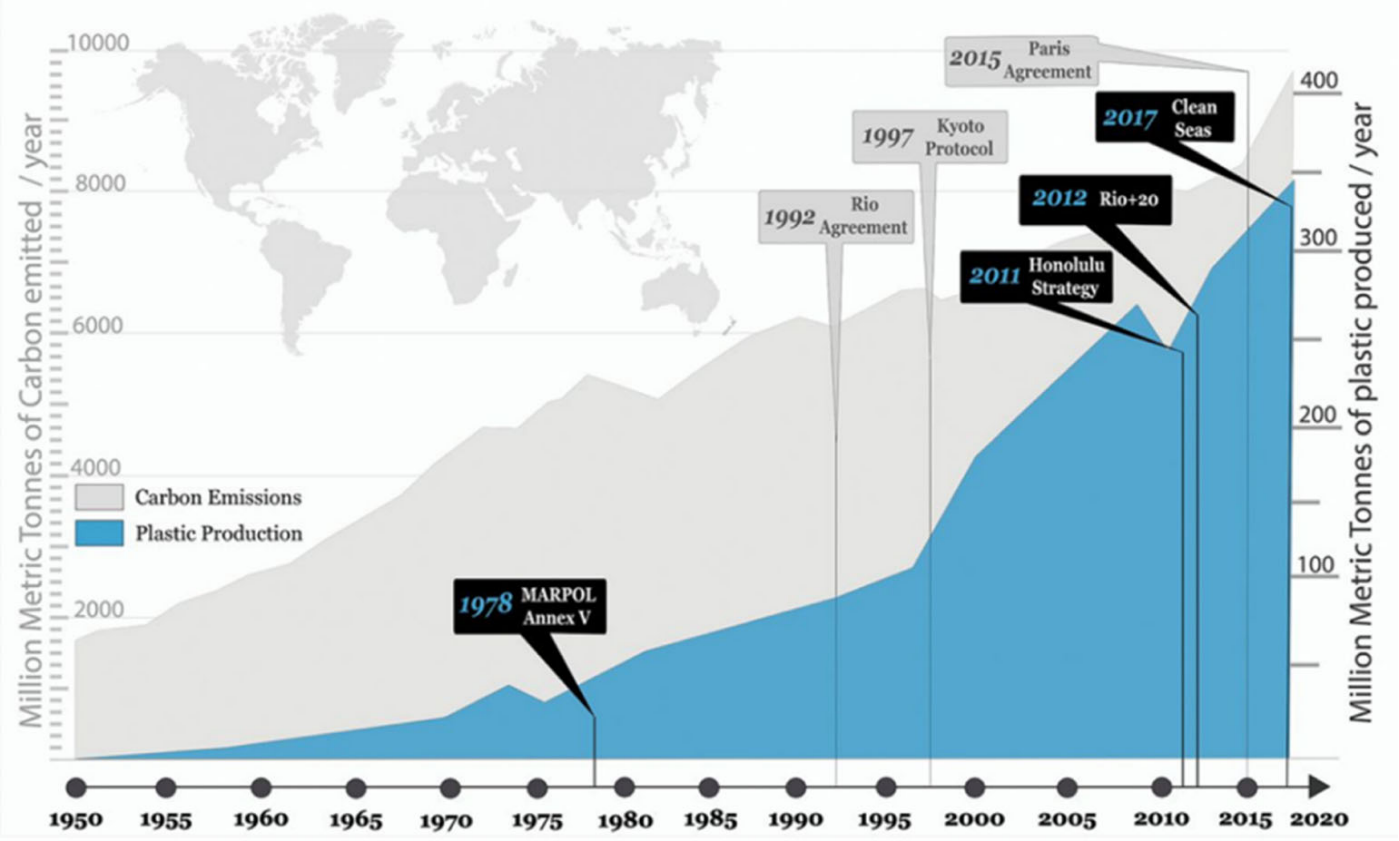
Accelerating rate of global plastic production relative to carbon emissions, 1950–2020. Taken from Borrelle *et al*. (2017).

The period following the end of World War II (1950s onward) was characterised by rapid industrial production, urban growth and the consumption of fossil fuels and other natural resources. Referred to as The Great Acceleration, changes in human activity during this period marks a fundamental shift in the state and functioning of the Earth's natural systems (Steffen *et al*., 2011) (Fig. 3). The scale of industrial growth during this period is exemplified by the emergence of densely packed metropolises – sprawling urban landscapes that replaced natural ecosystems (Steffen *et al*., 2015). For example, suburbanisation took hold in North America during the 1950s as families moved to the periphery of cities (Ellis, 2011). This shift led to changes in lifestyles, including the dependence on personal vehicles for travel. Urbanisation fuelled both economic growth and environmental degradation, leading to habitat loss, increased waste production and rising pollution levels, including increased greenhouse gas emissions, which drove climate change and its associated impacts (Foley *et al*., 2005; Steffen *et al*., 2011). Important aspects of this mid‐century industrial acceleration include the Green Revolution, which altered farming practices worldwide through the incorporation of high‐yield crops, synthetic fertilisers and pesticides (Foley *et al*., 2005). While increased food production during this time averted hunger and raised incomes for millions of people, it also caused severe soil degradation, over‐extraction of water for irrigation and waterway pollution (Tilman *et al*., 2002). Additionally, the invention and mass production of plastic from fossil fuels led to the proliferation of materials that were low cost, lightweight and durable and could be used in a wide array of applications (Geyer, Jambeck & Law, 2017). This convenience fundamentally altered consumption patterns, fostering a throwaway culture that prioritised convenience over sustainability. The impact that human activity had during this short period is illustrated by the millions of tons of plastic waste that enter terrestrial and marine ecosystems each year and cause damage to both humans and wildlife (Fig. 4) (Jambeck *et al*., 2015). More recently, the computer revolution of the 1980s and the social media revolution of the 2010s have created virtual environments that humans now spend increasing amounts time in at the expense of engagement with the natural world (Fotheringham, Wonnacott & Owen, 2000).

**Fig. 5:**
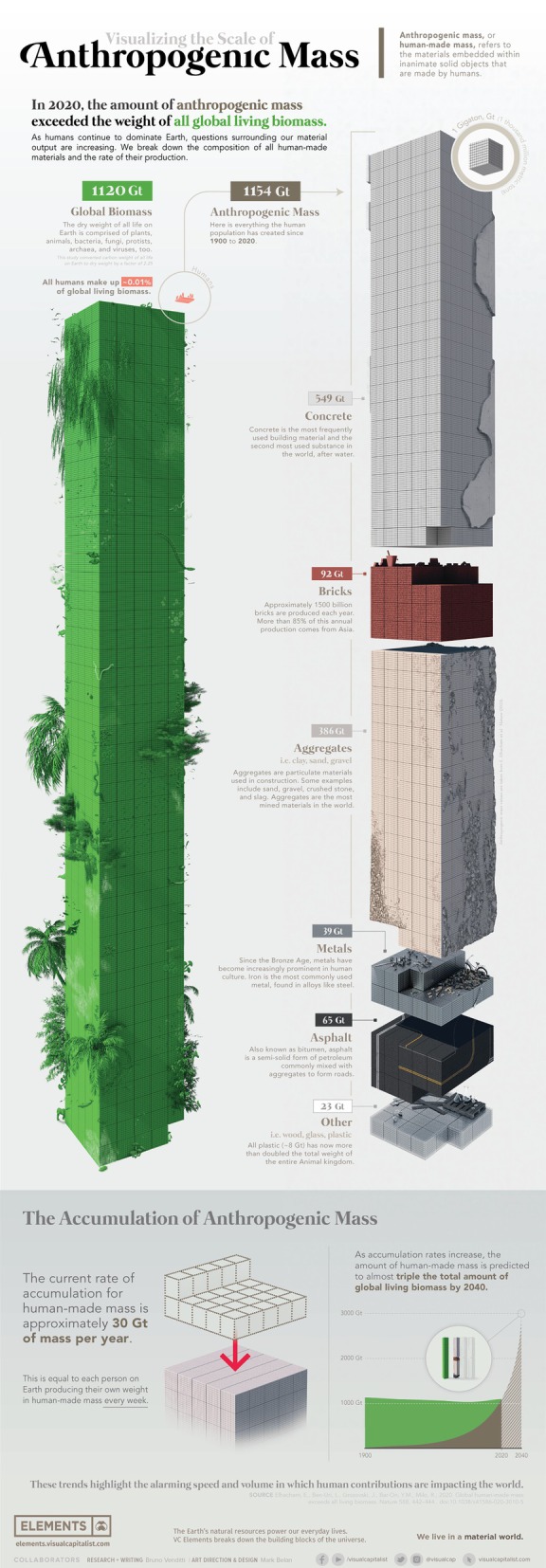
Visualising anthropogenic mass *versus* biomass in 2020 (adapted from https://elements.visualcapitalist.com/visualizing‐the‐accumulation‐of‐human‐made‐mass‐on‐earth/).

Over the past 70 years, the need to provide food, fibre, water and shelter to an ever‐growing human population has had profound effects on forests, farmlands, waterways and air (Foley *et al*., 2005). Croplands, pastures and urban areas have expanded alongside increased consumption of energy, water and fertiliser (Goldewijk, 2001). These changes have altered global biogeochemical cycles and diminished the ability of ecosystems to deliver services critical to human health and wellbeing (e.g. clean air and water) (Haberl *et al*., 2007). By the early 21st century, human activity had become the dominant geomorphological force on the planet (Cooper *et al*., 2018). Reflective of this impact, in 2020 anthropogenic (human‐made) mass (e.g. brick metal, plastic, glass) surpassed all global living biomass for the first time (Elhacham *et al*., 2020) (Fig. 5). This unprecedented resource exploitation has caused extensive habitat loss and is responsible for the current mass extinction of animal and plant species and the subsequent destabilisation of ecosystems (Dirzo *et al*., 2014). In many places this defaunation of the planet has undermined the capacity of ecosystems to sustain food production, maintain fresh water and regulate climate and air quality (Foley *et al*., 2005).

## III. THE EFFECT OF INDUSTRIALISATION ON EVOLUTIONARY FITNESS

Industrialisation has rapidly and profoundly transformed the various natural habitats of *Homo sapiens*. While industrialisation has provided numerous benefits, the resulting environmental changes may be imposing selective pressures (e.g. from air pollution, microplastics, artificial noise and light) that negatively impact evolutionary fitness (the ability to survive and reproduce).

**Fig. 6:**
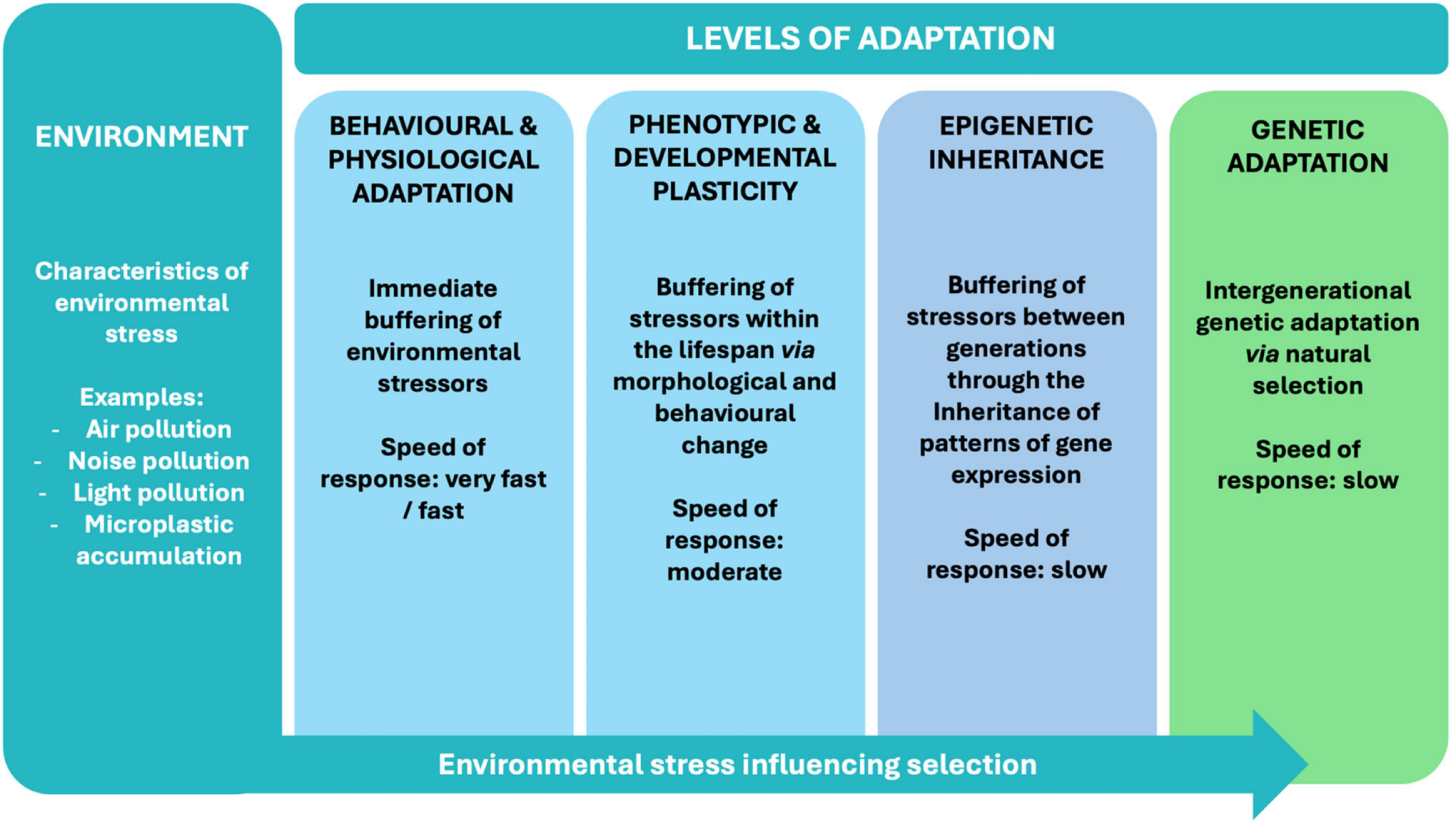
Hierarchical adaptative responses of humans to environmental stressors. Adaptation occurs across multiple timescales to maintain homeostasis and optimise evolutionary fitness. Immediate responses include behavioural and physiological adjustments. Over longer periods, phenotypic and developmental plasticity enable within‐lifetime adaptation. Across generations, epigenetic inheritance modifies gene expression in response to parental environments. If these adaptive mechanisms fail to buffer environmental stressors and preserve function, natural selection favours heritable traits that enhance survival and reproduction, leading to long‐term evolutionary change. Modified from Stock (2008).

Adaptation is the process by which phenotype is adjusted to optimise evolutionary fitness within a particular environment (Stock, 2008). Adaptation occurs through various processes, each operating on different timescales (Stock, Will & Wells, 2023) (Fig. 6). Immediate responses to environmental stressors include behavioural (e.g. seeking shade in response to heat) and physiological adaptation (e.g. sweating), which function as rapid, short‐term adjustments. Over longer timescales, adaptation *via* phenotypic plasticity (e.g. musculoskeletal adaptation to novel physical activities) or developmental plasticity (e.g. stature influenced by childhood nutrition) enable individuals to adjust to environmental challenges within a lifetime (Pigliucci, Murren & Schlichting, 2006; Nettle & Bateson, 2015). Across generations, epigenetic inheritance modifies patterns of gene expression in response to parental environments (e.g. reduced metabolic rate in offspring whose parents experienced a nutritionally deficient environmental deficit) (Lacal & Ventura, 2018). Each of these adaptative processes help to buffer the effects of environmental stressors on human biology without requiring underlying genetic change (Stock, 2012). This buffering facilitates homeostasis – the self‐regulating processes that maintain internal physiological stability despite external perturbation – thereby preserving biological function and evolutionary fitness. However, if this adaptive buffering fails to maintain homeostasis and function, adaptation *via* natural selection drives long‐term evolutionary change by favouring heritable traits that enhance survival and reproduction, leading to population‐level adaptation (Darwin, 1859). Humans, therefore, possess a hierarchical adaptive system that responds to environmental stressors to preserve homeostasis, function and evolutionary fitness (Fig. 6).

How might one assess the impact of novel environmental pressures associated with industrialisation on the fitness of contemporary humans? The Industrial Revolution began in the late 1700s, enough time for only eight or nine generations to pass, and there has been time for just three generations since industrialisation accelerated markedly after the mid‐20th century. Strong signals of natural selection (i.e. genetic adaptation *via* changes in allele frequency) would not be expected within such a short period of time. For comparison, the selective sweep of lactase persistence – one of the most significant genetic adaptations in the past 30,000 years of the evolutionary journey of *Homo sapiens* – took thousands of years to become widespread (Ségurel & Bon, 2017).

The absence of genetic evidence for natural selection, due to the short timeframe described above, offers little insight into the impact of industrialisation on human evolutionary fitness. However, examination of how industrialisation affects key functions that underpin fitness may be more revealing – particularly if functional impairment occurs during pre‐ and peri‐reproductive years when these functions directly influence survival and reproductive success, and therefore evolutionary fitness. Impairment of key functions would suggest that the adaptive mechanisms described above (Fig. 6) are insufficiently buffering environmental stressors and failing to maintain biological function, indicating impaired fitness. In this section, we consider the four key functions most likely to influence human evolutionary fitness – reproductive, immune, cognitive and physical function. Specifically, we assess first the influence of each of these four functions on human evolutionary fitness and then the impact of industrialisation on each function.

### (1) Industrialisation and reproductive function

#### (a) How does reproductive function influence evolutionary fitness?

From an evolutionary perspective, the primary goal of life is to pass on genes to the next generation through reproduction. Reproductive function, therefore, is *the* key determinant of evolutionary fitness, with all other biological functions supporting this objective. Reproductive success is influenced by a range of factors, including the desire and ability to attract mates, fecundity (i.e. the ability to reproduce), the longevity of reproductive potential, the ability to raise offspring to maturity and the provision of support to grandchildren. Any factors that affect sexual desire, attractiveness, fecundity, longevity or the ability to support the development of descendants may influence reproductive success and, consequently, evolutionary fitness.

#### (b) Is reproductive function impaired in industrialised environments?

**Fig. 7:**
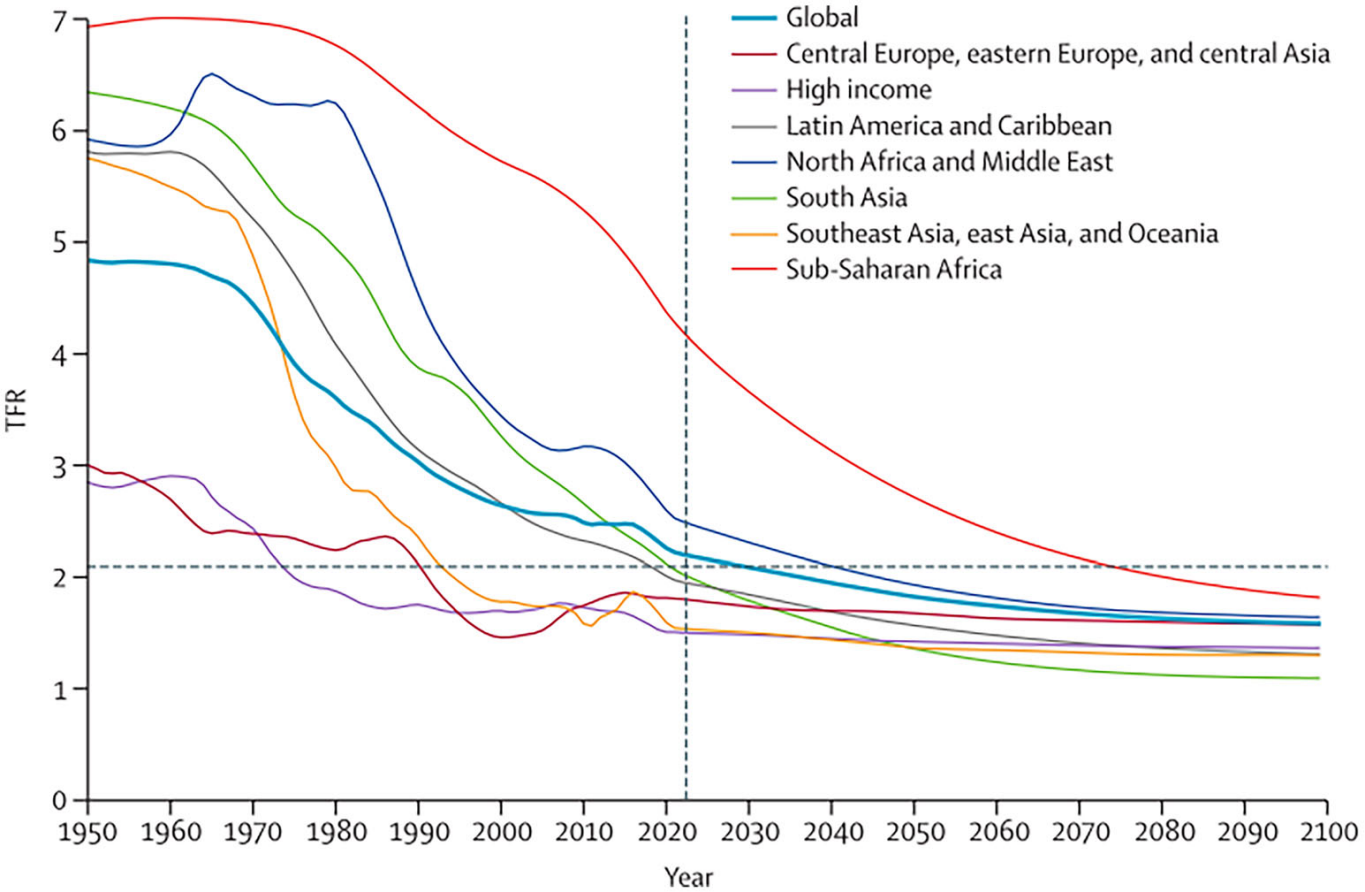
Declining global fertility rates. TFR = total fertility rate, horizontal dashed line represents replacement TFR. The vertical dashed line is the first forecast year of 2022 (Bhattacharjee *et al*., 2024).

As industrialisation continues to accelerate, global fertility rates are rapidly declining (Roser, 2014). Today, two‐thirds of the world's population live in countries with below‐replacement fertility (United Nations, 2022) (Fig. 7). While multiple factors drive this trend – such as delayed childbearing due to increased female access to education and professional careers, changing social norms and improved contraception – biological mechanisms linked to industrialisation appear to play a significant role (Skakkebæk *et al*., 2022).

A primary consequence of industrialisation is the release of environmental pollutants. Industrial activities like mining, construction, farming and energy production release billions of tonnes of chemically active materials annually, leading to widespread contamination. These pollutants are so pervasive that carcinogenic components are found in the blood, breast milk and tissues of all human populations, including infants and the unborn (Naidu *et al*., 2021).

Air pollution, which is significantly higher in urban areas (OECD, 2018), is also associated with impaired reproductive function. Exposure to elevated levels of air pollutants negatively affects both male and female gametogenesis (Carré *et al*., 2017) and increases the risk of spontaneous pregnancy loss (Leiser *et al*., 2019). Between 1973 and 2018, rising air pollution was associated with a 51.6% decline in global sperm concentration and a 62.3% decrease in total sperm count (Levine *et al*., 2023). A recent meta‐analysis further supports this link, associating air pollution with decreased sperm concentration, total sperm number and mobility (Xu *et al*., 2023).

In rural areas, industrial pesticides and herbicides impair reproductive function. A systematic review and meta‐analysis of 20 studies, for example, found a clear relationship between exposure to both organophosphate and *N‐*methyl carbamate insecticides and reduced sperm concentration (Ellis *et al*., 2023). Similarly, a recent systematic review found glyphosate‐based herbicides – the world's most widely used pesticides – were linked to lower testosterone, impaired sperm count and mobility, preterm birth (reported in two of three human studies), and birth defects (found in four of five studies, including two involving humans) (Mendez, Ordoñez‐Betancourth & Abrahams, 2022).

In parallel, unprecedented production of plastic products has led to widespread environmental contamination by micro‐ and nanoplastics – plastic fragments smaller than 5 mm and 1 μm, respectively – generated from the breakdown of objects such as tyres and clothes and which are directly manufactured for use in cosmetics. Their small size and persistence allows them to accumulate in body tissues *via* ingestion, inhalation and cutaneous absorption. Micro‐ and nanoplastics impair male fertility by decreasing sperm quality *via* inflammation and oxidative stress (D'Angelo & Meccariello, 2021) and disrupt female fertility by altering metabolism, inducing oxidative stress, damaging DNA and modulating the epigenome, and inducing developmental abnormalities in offspring (Geng *et al*., 2023). A recent review called for larger observational studies to evaluate the direct impact of micro‐plastics on human reproductive function (Hunt *et al*., 2024).

Chemicals leaching from industrial and consumer products also compromise reproductive function. Flame‐retardant chemicals such as polybrominated diphenyl ethers can permanently impair male reproductive function (Hales & Robaire, 2020). Similarly, phthalates – ubiquitous plasticisers found in consumer products – disrupt endocrine function, interfere with nuclear and membrane receptors, intracellular signalling pathways and gene expression associated with reproductive function and cause fertility disorders in both men and women (Hlisníková *et al*., 2020).

In summary, worldwide fertility rates are declining, partly due to the impairment of reproductive function caused by industrial pollution. Air pollution, more pronounced in urban settings, disrupts gametogenesis, reduces sperm quality and increases risk of pregnancy loss. In rural areas, pesticide and herbicide exposure lowers sperm quality and testosterone while increasing risks of birth defects and preterm birth. Micro‐ and nanoplastics accumulate in body tissues, decreasing sperm quality in men and impairing reproductive function in women. Additionally, chemicals such as phthalates and flame retardants disrupt the endocrine system, further interfering with reproductive pathways and leading to fertility disorders.

In addition to these direct effects, industrialisation may indirectly impair reproductive success. As discussed in Section III.2, environmental industrialisation can compromise immune function, potentially reducing the capacity of older individuals to care for descendants. Given that intergenerational support – such as grandparental care (Sear & Mace, 2008) – can enhance reproductive success, such impairments may have broader implications for evolutionary fitness. These combined effects suggest that industrialisation poses a potential threat to human reproductive function and evolutionary fitness.

### (2) Industrialisation and immune function

#### (a) How does immune function influence evolutionary fitness?

Immune function is critical for both survival and reproduction, making it a key component of evolutionary fitness. The immune system protects against pathogens, harmful environmental substances and cellular changes such as tumour formation. Inadequate immune function can therefore directly reduce fitness by compromising survival. Additionally, immune function affects reproductive success as illness can disrupt reproductive processes (Souho, Benlemlih & Bennani, 2015; Farsimadan & Motamedifar, 2020), reduce attractiveness to mates (Oaten, Stevenson & Case, 2009), and impair resource acquisition (Hoving *et al*., 2010) and the capacity of older individuals to care for descendants. Thus, immune function supports both survival and reproduction, directly influencing evolutionary fitness.

#### (b) Is immune function impaired in industrialised environments?

Observational research, controlling for factors such as demographic and socioeconomic characteristics, suggests that environmental industrialisation impairs immune function, increases infection susceptibility and contributes to chronic inflammatory conditions such as allergies, autoimmune diseases and inflammatory bowel disease (Rook, Lowry & Raison, 2013). For example, a study of 345,143 Dutch residents linked lower neighbourhood greenness (a proxy for industrialisation) with higher incidences of upper respiratory tract, intestinal and urinary tract infections (Maas *et al*., 2009). Similarly, a nationwide US study found lower green space availability correlated with higher SARS‐CoV‐2 infection rates (Jiang *et al*., 2022). However, a separate Chinese study found mixed results – dysentery showed negative correlations with neighbourhood greenness, while tuberculosis and malaria showed positive correlations (Liu *et al*., 2019).

Experimental studies have also reported immune function impairment in industrialised environments. Systematic reviews have noted increased proinflammatory and decreased anti‐inflammatory cytokine levels in more industrialised settings relative to more natural environments (Andersen, Corazon & Stigsdotter, 2021; Chae *et al*., 2021), although confidence in these conclusions is limited by small sample sizes and other methodological weaknesses. A more rigorous experimental approach has demonstrated a negative impact of industrialisation on natural killer (NK) cell function – a type of white blood cell that destroys infected and cancerous cells. Demonstration of increased NK cell number, activity and anti‐cancer protein levels following a forest visit was later supported by *in vitro* (Li, Miyazaki & Krensky, 2006) and *in vivo* investigations (Li *et al*., 2009) that identified phytoncides – volatile organic compounds released by trees – as the compound responsible for the changes (albeit with a limited sample size of 12). More broadly, two complementary mechanisms may explain the observed impairment of immune function in industrialised environments.

First, contemporary populations in more industrialised environments may be losing contact with environmental microorganisms essential for effective immunoregulation. The Old Friends Hypothesis (Rook, 2012, 2013), building on the now‐revised Hygiene Hypothesis (Strachan, 1989), argues that ancestral exposure to microorganisms from soil, animals and faeces promoted immunoregulation and inhibited damaging immune reactions such as excess inflammation (Scudellari, 2017). Infection by helminths soon after birth, for example, is powerfully immunomodulatory and provides protection against autoimmune diseases such as multiple sclerosis (Fleming *et al*., 2011). Modern sanitation, however, reduces exposure to the microbiota with which our immune systems coevolved (Rook, 2012; Smith *et al*., 2019), contributing to immune dysregulation and the increased prevalence of autoimmune and allergic diseases (Blaser, 2017). The Biodiversity Hypothesis (Haahtela *et al*., 2013) expands on the ideas introduced by the Hygiene and Old Friends hypotheses [as well as the Microbial Diversity (Matricardi, 2010) and Microbial Deprivation hypotheses (Björkstén, 2012)], positing that early‐life exposure to diverse environmental microorganisms is crucial for developing a healthy microbiome. Supporting this, residents of low‐biodiversity areas have reduced skin microbial diversity (Hanski *et al*., 2012), which is closely linked to immunoregulation *via* increased plasma transforming growth factor β1 levels, the proportion of regulatory T cells and the ratio of interleukin‐10 to interleukin‐17 (Roslund *et al*., 2020; VanEvery *et al*., 2023).

Second, aspects of more industrialised environments to which we are not (yet) well adapted may impair immune function. Air pollution, for example, directly negatively impacts a range of immune cells, disrupting antimicrobial immune responses, triggering pro‐inflammatory responses and intensifying T‐helper lymphocyte types 2 and 17 adaptive immune responses, as seen in asthma and chronic obstructive pulmonary disease exacerbations (Glencross *et al*., 2020). Anthropogenic noise, such as that produced by traffic, impairs immune function *via* sleep disruption, which suppresses antiviral responses and increases systemic inflammation (Irwin, 2012) and chronic stress, which disrupts innate and adaptive immunity (Prasher, 2009; Recio *et al*., 2016). Nighttime artificial light can disrupt circadian regulation of immune function (Walker *et al*., 2022). For example, a recent randomised crossover study reported that sleeping with a light on increased inflammation, as indicated by elevated plasma high‐sensitivity C‐reactive protein levels (Mindel *et al*., 2019). Additionally, animal models show that air, noise and light pollution perturb the liver transcriptome, suppressing genes linked to innate immunity and antioxidant activity while upregulating tumorigenesis‐related genes (Isaksson *et al*., 2024).

In summary, observational and experimental evidence highlights the link between environmental industrialisation, impaired immune function and decreased fitness by increasing susceptibility to infection, chronic inflammation and cancer. Two key mechanisms have been proposed to explain this immune impairment: (*i*) immune dysregulation arising from decreased contact with environmental microorganisms; and (*ii*) environmental stressors such as air pollution, noise, and artificial light, which impair immune cell function, promote inflammation and disrupt circadian‐regulated immune responses. These factors collectively suggest that industrialisation can negatively impact immune function and potentially impact evolutionary fitness.

### (3) Industrialisation and cognitive function

#### (a) How does cognitive function influence evolutionary fitness?

The development of an enlarged and elaborate brain is considered a defining characteristic of our species (Lee & Wolpoff, 2003). This encephalisation has enabled humans to thrive within their own socio‐cognitive niche, which requires advanced cognitive functioning (Whiten & Erdal, 2012). The pronounced encephalisation that accompanied the evolution of the *Homo* clade enhanced individual fitness *via* a plethora of benefits including the development of complex social strategies (Byrne & Corp, 2004), improved food acquisition (Gibson, 1986) and innovative problem solving (Reader & Laland, 2002). These traits have increased survival and reproductive success, contributing to a greater life expectancy at first reproduction (Barrickman *et al*., 2008).

In the 21st century, cognitive function still correlates positively with various fitness indicators, including physical health and longevity (Gottfredson & Deary, 2004), resource acquisition and socioeconomic status (Gottfredson & Deary, 2004; Nettle & Pollet, 2008) and biological markers such as fluctuating asymmetry (Banks, Batchelor & Mcdaniel, 2010) and male sperm quality (Arden *et al*., 2009). Cognition may therefore serve as a heritable indicator of an individual's ability to survive adaptive challenges (Miller, 2000), as evidenced by its high value in both male and female short‐ and long‐term mate selection (Jonason *et al*., 2019). Conversely, compromised cognition reduces fitness *via* mechanisms including poorer mental (Chen *et al*., 2006) and physical health (Frisoni *et al*., 2000), functional impairments (Auyeung *et al*., 2008), decreased ability to interact socially and develop relationships (Duclos *et al*., 2018) and reduced attractiveness to potential sexual partners (Miller, 2000). Although cognitive function is inversely correlated with fertility – more strongly so in women than men – this association is largely driven by education (Retherford & Sewell, 1989) and delayed childbearing (Woodley *et al*., 2019).

#### (b) Is cognitive function impaired in industrialised environments?

Large‐scale observational data suggest that environmental industrialisation has a negative impact on cognitive function. For example, a one‐year study of ~2600 children (aged 7–10 years) in Barcelona, Spain, found that the degree of neighbourhood industrialisation (as assessed *via* the normalised difference vegetation index, NDVI) was associated with impaired executive function development after controlling for factors such as socioeconomic status (Dadvand *et al*., 2015). Similarly, longitudinal data from low‐income families revealed that children of families relocating to greener neighbourhoods (i.e. with decreased neighbourhood industrialisation) exhibited greater cognitive improvements than those moving to less‐green neighbourhoods (Wells, 2000). Among adults, residential proximity to predominantly natural environments has been linked to slower age‐related cognitive decline across multiple cognitive domains, independent of social cohesion and physical activity (de Keijzer *et al*., 2018). A similar pattern was observed in a survey of 15,874 older adults (aged 65+ years) randomly selected from 154 Chinese cities, which revealed a negative relationship between the rate of city urbanisation and performance in tests of orientation, memory, language, calculation, attention and recall (Tian *et al*., 2024).

These observational data are complemented by experimental investigations that have reported impaired cognition following exposure to more industrialised environments. For example, studies conducted in Asia and North America that took participants for 50–55‐min walks in highly industrialised urban centres observed suppression of attention, working memory, creativity, problem solving, sequencing, visual scanning and executive function performance relative to comparable walks in forested areas (Berman, Jonides & Kaplan, 2008; Shin *et al*., 2011). Recent systematic reviews and meta‐analyses support these findings, highlighting impairment of attention, working memory and cognitive flexibility following exposure to industrialised environments in both children and adults, relative to exposure to more natural environments (Ohly *et al*., 2016; Nguyen & Walters, 2024).

The mechanisms that link industrialisation and impaired cognition are yet to be fully elucidated, although air pollution may be a key factor (Drew, 2025). Polluted air is associated with poorer performance in verbal and mathematical tests, with effects becoming more pronounced with age, particularly for men with lower levels of education (Zhang, Chen & Zhang, 2018). A systematic review of 31 studies from the Americas, Asia and Europe found consistent links between air pollution and cognitive impairment across the life course, including neurodevelopmental delays in younger children, reduced academic and neurocognitive performance in older children and accelerated cognitive decline in adults (Clifford *et al*., 2016). Similarly, indoor air quality also influences cognition. For example, increases in airborne fine particulate matter significantly increases chess players' likelihood of making unfavourable moves (Künn, Palacios & Pestel, 2023).

Industrial soundscapes and visual aspects of industrialised environments can also impair cognition. Observational and experimental data have demonstrated that workplace and vehicle noise, for example, impairs worker productivity and efficiency (Qutubuddin, Hebbal & Kumar, 2012). Similarly, a review of 31 studies reported impaired attention, working memory, impulse inhibition and episodic recall in individuals exposed to urban noise stress (Wright *et al*., 2014). In parallel, experimental studies show that listening to sound recordings of industrial environments disrupts directed attention (the ability to voluntarily control the focus of thoughts) relative to nature sounds (Longman *et al*., 2025; Van Hedger *et al*., 2019). Furthermore, viewing pictures of industrialised scenes can reduce directed attention relative to pictures of natural scenes (Berman *et al*., 2008).

In summary, cognitive function plays a crucial role in evolutionary fitness, underpinning survival and reproductive success through enhanced social strategies, resource acquisition and problem‐solving abilities. Today, cognitive function correlates with multiple fitness indicators, including health, socioeconomic status and mate selection, while compromised cognition is linked to poorer mental and physical health, social difficulties, and reduced attractiveness to potential mates. Emerging evidence suggests that industrialised environments have a negative effect on cognitive function, with large‐scale observational studies associating urbanisation with slower cognitive development in children and accelerated decline in adults. Experimental research further demonstrates that exposure to industrial settings suppresses attention, memory and executive function through mechanisms including air pollution, noise, and visual stressors.

### (4) Industrialisation and physical function

#### (a) How does physical function influence evolutionary fitness?

Physical function, defined here as the ability to perform tasks requiring strength and/or cardiovascular fitness, is crucial for survival and reproduction. In non‐industrial societies, physically challenging activities like hunting and fighting contribute to both intra‐ and intersexual selection. Hunting requires endurance (Lieberman *et al*., 2006; Liebenberg, 2006) and upper body strength (Apicella, 2014), and likely signalled male evolutionary fitness, enhancing sexual attractiveness and promoting reproductive success (Kaplan & Hill, 1985; Hill, Kaplan & Hawkes, 1993; Marlowe, 2004; Longman, Wells & Stock, 2015) – although the extent to which hunting ability was directly shaped by sexual or natural selection remains a topic of debate. Much of the literature has focussed on male physical capabilities and involvement in hunting, often portraying female contributions to subsistence as primarily centred on gathering. However, recent analyses question the robustness of this rigid sexual division of labour. Both archaeological (Lacy & Ocobock, 2024) and physiological evidence (Ocobock & Lacy, 2024) challenge the assumption that females did not – or could not – also participate meaningfully in hunting. Moreover, subsistence tasks commonly associated with female roles, such as gathering and extractive foraging using tools such as digging sticks, as well as other recurrent physical activities like infant carrying, required strength and endurance and likely contributed to adaptive responses (Stock & Pfeiffer, 2001; Wall‐Scheffler, 2022).

Similarly, fighting played a significant role in male competition for resources and defence, with greater upper body strength being selectively favoured (Gallup & Fink, 2018). There is substantial support for the concept that sexual selection has favoured male traits associated with fighting ability, such as upper body strength (Lassek & Gaulin, 2009), craniofacial robustness (Carrier & Morgan, 2015), aggression (Wrangham, 2017) and the capacity visually to assess formidability (Sell *et al*., 2009).

Physical function continues to influence evolutionary fitness today. Aerobic fitness and strength are key indicators of resilience, long‐term health and disease risk in both men and women (Silverman & Deuster, 2014). Physical fitness also impacts sexual selection; male muscularity signals mate quality (Durkee *et al*., 2019) and correlates positively with both number of sexual partners and – in societies lacking widespread access to contraception – number of offspring (Frederick & Haselton, 2007; Lidborg, Cross & Boothroyd, 2022).

Importantly, physical function contributes to social status, which in turn influences reproductive success. Across a range of animal species, including *Homo sapiens*, males with higher social status tend to have greater reproductive success (Hopcroft, 2006; Nettle & Pollet, 2008). In both non‐industrial and industrialised societies, physical strength, endurance and athletic ability are often linked to status. In contemporary industrialised societies, sporting competitions often serve as formalised contests for status (Edwards, 2006), with success conferring both monetary and symbolic rewards, increasing mating opportunities and reflecting ongoing intrasexual selection (Buss, 1989).

Research considering the link between female physical fitness and sexual selection, however, remains limited (Hönekopp, Bartholomé & Jansen, 2004). Existing studies suggest that physical fitness may improve pregnancy outcomes for women (Hönekopp *et al*., 2004). Nevertheless, while body shape and size have been linked to reproductive success in females (Jasieńska *et al*., 2004), the role of physical performance in sexual selection remains underexplored, indicating a need for further investigation.

#### (b) Is physical function impaired in industrialised environments?

Large‐scale observational studies suggest that industrialisation negatively impacts endurance and strength. In children and adolescents, cardiovascular fitness and upper body strength are consistently lower in urban residents compared to rural areas across various countries (Machado‐Rodrigues *et al*., 2014; Sylejmani *et al*., 2019). Among adults, low greenness in industrialised areas is linked to weaker handgrip strength (Feng *et al*., 2023) and older adults living in industrialised areas show greater handgrip weakness and a faster decline in physical function in both walking speed and handgrip strength (de Keijzer *et al*., 2019). However, the degree to which relevant covariates were controlled for in these studies is variable.

Air pollution is a key factor in the impairment of physical function in industrialised areas. Industrial pollutants such as airborne particulate matter, ozone, carbon monoxide, sulphur dioxide and nitrogen dioxide negatively impact pulmonary and cardiovascular function (Chen & Kan, 2008), which impairs endurance performance (Giles & Koehle, 2014). For example, observational data have linked higher concentrations of airborne particulate matter with slower marathon times in women (Marr & Ely, 2010) and decreased 5000‐m performance in men, even when below the US Environmental Protection Agency's threshold for good air quality (Cusick, Rowland & DeFelice, 2023). Complementary experimental studies have reported reduced work capacity in the presence of particulate matter (Cutrufello *et al*., 2011). Similarly, ozone exposure causes respiratory discomfort (Adams & Schelegle, 1983) and is associated with decreased performance in track‐and‐field endurance events (Mullins, 2018), and reduced oxygen consumption, exercise duration and overall performance (Foxcroft & Adams, 1986). Carbon monoxide, a prevalent urban pollutant, also reduces oxygen consumption and exercise capacity (Flouris *et al*., 2012). More broadly, air pollution is linked to decreased hand‐grip strength (Lin *et al*., 2020), increased risk of sarcopenia (i.e. age‐related loss of muscle mass, strength and function) (Zhang *et al*., 2023) and reduced predicted maximal oxygen uptake (*V*O_2max_) and cardo‐respiratory fitness in children (Gao *et al*., 2013).

In summary, in non‐industrial societies, endurance and upper‐body strength played key roles in hunting, combat and resource competition, influencing both intra‐ and intersexual selection by signalling mate quality and enhancing reproductive success. In contemporary industrialised societies, physical fitness remains linked to resilience, long‐term health and social status, with male muscularity correlating with mating success and athletic competitions serving as modern status contests. While research on female physical fitness and sexual selection is limited, evidence suggests that physical fitness may improve pregnancy outcomes and overall reproductive success. Observational and experimental evidence suggests that environmental industrialisation may compromise physical function, as reflected by urban populations exhibiting lower cardiovascular fitness and strength. Environmental factors such as air pollution appear to mediate this decline in performance, potentially reducing evolutionary fitness.

### (5) Industrialisation and chronic stress

Chronic stress, while not a direct determinant of evolutionary fitness, is relevant for two reasons. First, evolutionary theory suggests that removing a population from the environment to which it is primarily adapted and exposing it to novel challenges may induce stress. Increased prevalence of chronic stress in more industrialised environments would therefore suggest that such environments pose an adaptive challenge. Second, chronic stress can itself indirectly reduce evolutionary fitness by impairing reproductive, immune, cognitive and/or physical function.

While adaptive in acute contexts, prolonged stress harms physiological and psychological health. Chronic stress contributes to hypertension – the leading global disease risk factor (Lim *et al*., 2012) – and is associated with wide‐ranging physical and psychological conditions including cardiovascular disease, metabolic disorders, musculoskeletal issues and mental health impairments (Chrousos, 2009). Chronic stress also disrupts key physiological functions (Sapolsky, Romero & Munck, 2000).

#### (a) How does stress influence evolutionary fitness?

Chronic stress suppresses reproductive function in both sexes by disrupting the hypothalamo–pituitary–gonadal axis, reducing gonadotropin releasing hormone (GnRH) secretion in the hypothalamus, luteinising hormone (LH) and follicle stimulating hormone (FSH) secretion in the pituitary and disrupting steroidogenesis and/or gametogenesis in the gonads (Whirledge & Cidlowski, 2010). Stress reduces female fecundity by disrupting the hypothalamic–pituitary–ovarian (HPO) axis, as evidenced by a 29% decrease in conception rates among highly stressed women (Lynch *et al*., 2014). In men, stress reduces spermatogenesis and sperm quality (Ilacqua *et al*., 2018), potentially through reductions in LH and testosterone pulsing (Gollenberg *et al*., 2010; Corona *et al*., 2016). Chronic stress can also reduce sexual desire and arousal in both sexes, although this is less studied in men (ter Kuile, Vigeveno & Laan, 2007; Nimbi *et al*., 2020).

While acute stress may temporarily stimulate an immune response, serving to restore homeostasis, chronic stress suppresses both innate and adaptive immunity by disrupting the balance between Type 1 and Type 2 cytokines, increasing chronic inflammation and impairing immune cell activity. This suppression increases vulnerability to physical and psychological illnesses including autoimmune diseases, irritable bowel syndrome and schizophrenia (Morey *et al*., 2015). Chronic stress may also increase cancer risk by decreasing levels of Type 1 cytokines and protective T cells, while enhancing those of regulatory/suppressor T cells (Dhabhar, 2014). Stress also promotes behavioural changes such as decreased sleep and diet quality, reduced exercise and increased smoking and drug use, further compromising immune function (Segerstrom & Miller, 2004; Glaser & Kiecolt‐Glaser, 2005).

Chronic stress can also impair cognitive function, including working memory, attention, response inhibition and cognitive flexibility (Girotti *et al*., 2018). Glucocorticoid overexposure may lead to neuronal loss, hippocampal dendrite atrophy, reduced hippocampal volume and dysregulation of the amygdala and prefrontal cortex (Vyas *et al*., 2016; Lupien *et al*., 2018).

In addition to cognitive effects, chronic stress impairs physical performance by disrupting hormonal, physiological and psychological processes. Stress‐induced hormonal imbalances decrease levels of anabolic hormones (e.g. testosterone, growth hormone, insulin‐like growth factor) and increase those of catabolic hormones (e.g. cortisol, adrenaline, cytokines), leading to muscular atrophy and impaired recovery (Schakman *et al*., 2009). Stress also disrupts energy balance by impairing hormonal action, glucose uptake and mitochondrial function (van der Kooij, 2020), reducing endurance performance. Decreased physical activity (Stults‐Kolehmainen & Sinha, 2014), disrupted sleep (Lo Martire *et al*., 2020) and greater susceptibility to illness due to immune suppression compound these effects (Dhabhar, 2014).

#### (b) Is stress higher in industrialised environments?

Observational and experimental studies have linked environmental industrialisation with increased chronic stress and related health conditions (Lederbogen *et al*., 2011). Early laboratory‐based studies found slower recovery from induced stress after viewing videos of industrialised urban environments compared to natural landscapes (Ulrich *et al*., 1991). More recent research consistently reports elevated hypothalamus–pituitary–adrenal (HPA) and sympathetic‐adreno‐medullar (SAM) axis biomarkers (including salivary cortisol, heart rate, heart rate variability, blood pressure and prefrontal cortex activity) following exposure to industrialised settings, relative to natural environments, in diverse populations (Frumkin *et al*., 2017; Hansen, Jones & Tocchini, 2017; Bratman *et al*., 2019). A prime example is a decade‐long Japanese study which found significant stress differences after just 15 min in industrialised *versus* natural environments (Song, Ikei & Miyazaki, 2016). Exposure to industrialised environments also increases psychological stress, suppressing mood and heightening depression, anxiety, rumination and negative affect, while reducing feelings of stress relief and revitalisation, relative to nature exposure (Frumkin *et al*., 2017; Hansen *et al*., 2017; Bratman *et al*., 2019).

Chronic stress, while not customarily considered to influence evolutionary fitness directly, therefore does suppress key functions that underpin survival and reproduction. In both sexes, chronic stress suppresses reproductive function – reducing conception rates in women and impairing sperm quality in men – while also compromising immune resilience and cognitive and physical performance. Observational and experimental studies link environmental industrialisation with higher rates of chronic stress, reflected by changes in various physiological biomarkers and psychological outcomes, while spending time in nature mitigates these effects, underscoring the role of environment in stress regulation and evolutionary fitness.

### (6) Summary of the effect of industrialisation on evolutionary fitness

While the ongoing process of environmental industrialisation has brought transformative societal benefits, it has also rapidly and significantly transformed our species' various natural habitats. Contemporary industrialised environments differ fundamentally from the range of natural ancestral habitats occupied by our hominin ancestors due to their lack of natural features linked to health (e.g. phytoncides) and, simultaneously, the imposition of novel environmental challenges (e.g. air pollution). Observational and experimental evidence highlights the negative effect of industrialisation on reproductive, immune, cognitive and physical function, as well as increased stress (which, in turn, impacts these key functions). Combined, this evidence highlights the negative impact of industrialisation on human fitness.

## IV. HAS INDUSTRIALISATION CREATED AN ENVIRONMENTAL MISMATCH IN *HOMO SAPIENS*?

The concept of evolutionary mismatch describes the adverse consequences arising from an imbalance between an organism and its environment. This disequilibrium can arise when the environment changes more rapidly than an organism can adapt, creating an adaptive lag (Gluckman & Hanson, 2006; Lloyd, Wilson & Sober, 2011; Brenner *et al*., 2015). In dynamic environments, mismatch is a key driver of evolutionary adaptation. The speed and magnitude with which *Homo sapiens* is capable of habitat transformation make evolutionary mismatches particularly relevant for our species, with potential negative implications for health and evolutionary fitness (Brenner *et al*., 2015; Gluckman, Hanson & Low, 2019; Gurven & Lieberman, 2020).

Here, we ask whether industrialisation has created a human‐induced environmental mismatch between our primarily nature‐adapted biology and the novel environmental challenges imposed by contemporary industrialised environments. This question stems from the observations presented above, that the various natural habitats of our species have undergone a rapid and profound transformation during the last 200–300 years. Ancestral environments were predominantly composed of naturally occurring features and materials, both organic (i.e. flora, fauna and microbiota) and inorganic (e.g. geological formations and water bodies). By contrast, contemporary environments are largely human made, with far fewer natural features (although a continuum exists between these two extremes) and introduce novel stressors such as pollution. In parallel, a growing body of empirical evidence highlights the detrimental effects of environmental industrialisation on key functions that underpin evolutionary fitness, as well as on stress regulation and general health. Mechanistically, this habitat change may impair contemporary human biology through two primary pathways: (*i*) aspects of industrialised environments, to which humans are not yet well adapted, present novel challenges that impair function and fitness; and/or (*ii*) contemporary humans have lost contact with essential aspects of the natural world that are necessary for optimal health, function and fitness.

**Fig. 8:**
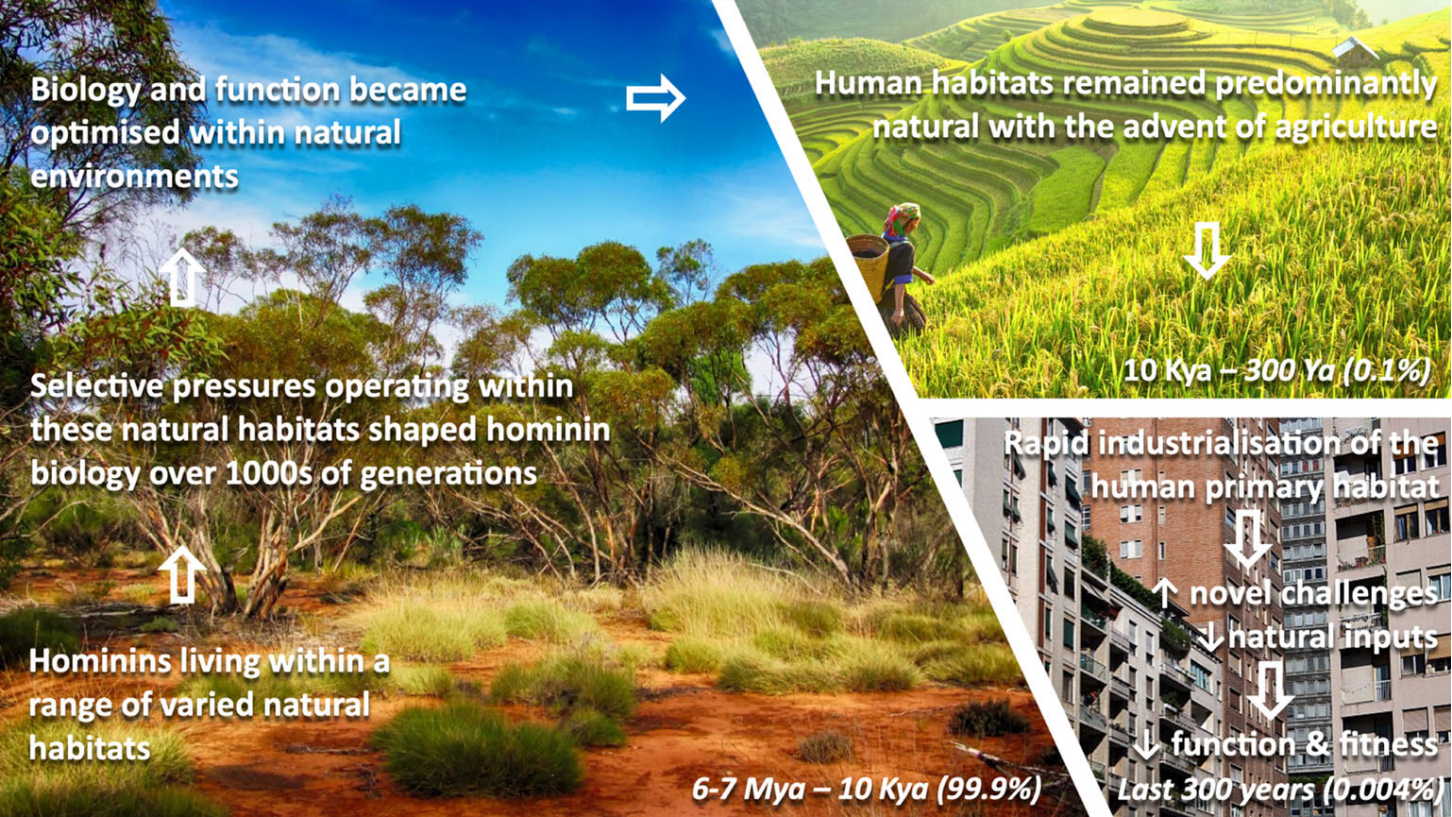
Visual representation of the Environmental Mismatch Hypothesis.

The rapid pace of change to our habitat, combined with mounting evidence of compromised function in industrialised environments, leads to the hypothesis that industrialisation has created an environmental mismatch. This Environmental Mismatch Hypothesis is depicted in Fig. 8, which visualises the potential misalignment between our primarily nature‐adapted biology and the novel challenges of modern industrialised environments.

## V. TESTING THE ENVIRONMENTAL MISMATCH HYPOTHESIS

Future investigation into whether industrialisation has created a human‐induced environmental mismatch would benefit from following three key principles.

First, research should comprehensively assess the impact of industrialised environments on key functions that underpin evolutionary fitness, relative to natural environments representative of ancestral habitats. Such an approach would reconcile the current scarcity of high‐quality, reproducible studies that consider how industrialisation affects function and evolutionary fitness. Second, studies should be designed to prolong exposure (lasting days rather than minutes) to both natural and industrial environments, allowing more time for environmental features to influence human biology. This would provide deeper insights into the cumulative effects of highly industrialised and natural environments and clarify how adaptive processes in ancestral environments have shaped human phenotypes. Regular assessment of biological function throughout exposure would further elucidate dose–response relationships. Third, research should explore the mechanisms by which industrialisation influences biological function (Lloyd *et al*., 2011). This can be achieved through a combination of field and laboratory experiments designed to identify specific aspects of industrialised environments that impair human function and features of ancestral natural habitats that may be absent from contemporary industrialised settings and are essential for optimal function.

## VI. IMPLICATIONS OF AN ENVIRONMENTAL MISMATCH

The question of whether global industrialisation has created a human‐induced environmental mismatch has broad interdisciplinary significance.

First, the large‐scale natural experiment provided by industrialisation and urban migration offers an opportunity to deepen our understanding of how *Homo sapiens* adapt to novel habitats to optimise fitness. Insights gained from this research could enhance understanding not only of the biological consequences of recent environmental industrialisation, but also of the adaptive mechanisms that enabled *Homo sapiens* to disperse across diverse ecological settings over the past 300,000 years. For example, contemporary urban migration provides a framework for examining how the human hierarchical adaptive system responds to environmental stressors to preserve homeostasis, function and evolutionary fitness. Experimentation designed to quantify how modern humans adapt to the challenges of urban migration may offer valuable analogues for understanding how ancestral populations responded to environmental challenges in novel ancestral environments, such as those created by changes in food availability or pathogen exposure associated with pronounced changes to the climate.

Industrialisation has been driven by short‐term financial gain, with little consideration of the long‐term impact of industrial activities for population or environmental health. The Environmental Mismatch Hypothesis therefore also has practical, forward‐facing applications, particularly in addressing pressing contemporary challenges. Urban living, with its associated stressors, poses significant physical and mental health risks (Segerstrom & Miller, 2004; Chrousos, 2009; Lederbogen *et al*., 2011). While countries such as Finland, South Korea and Japan promote nature‐based stress management (Frumkin *et al*., 2017; Hansen *et al*., 2017; Bratman *et al*., 2019), such practices remain underutilised elsewhere due to limited research into the mechanisms linking nature exposure to health benefits (Van den Berg, 2017). Understanding how industrialisation affects human biology could provide the evidence needed to support nature‐based therapeutic interventions.

The Environmental Mismatch Hypothesis is also relevant to global sustainability. Industrialisation has driven economic growth at the cost of severe ecological degradation (Whitmee *et al*., 2015), contributing to climate disruption, freshwater scarcity, biodiversity and habitat loss, pollution, poor soil microbial health and declining food security (Lovins *et al*., 2018). These crises now threaten human health, potentially reversing recent global health gains (Romanelli *et al*., 2015). Advancing research on nature's role in human health could help reframe natural environments as essential to human life, fostering stronger conservation and regeneration efforts.

## VII. CONCLUSIONS

(1) For the vast majority of the evolutionary journey of *Homo sapiens*, a range of natural environments defined the parameters within which selection shaped human biology.
(2) The rapid environmental changes of the Anthropocene, driven by industrialisation, have profoundly transformed the human habitat, imposing novel environmental pressures.
(3) The rate of environmental change may be outpacing human adaptive capacity, potentially compromising our evolutionary fitness.
(4) A growing body of empirical evidence suggests that environmental industrialisation negatively impacts human biology, suppressing key biological functions essential for survival, reproduction and, therefore, evolutionary fitness.
(5) We consider whether industrialisation has created a mismatch between our primarily nature‐adapted biology and the novel challenges imposed by contemporary industrialised environments, a possibility framed through the Environmental Mismatch Hypothesis.
(6) This mismatch could have broad interdisciplinary relevance, from advancing understanding of adaptational processes in prehistoric and 21st century humans to addressing contemporary public health challenges and issues of global sustainability.
